# Wnt Lipidation and Modifiers in Intestinal Carcinogenesis and Cancer

**DOI:** 10.3390/cancers8070069

**Published:** 2016-07-18

**Authors:** Elke Kaemmerer, Nikolaus Gassler

**Affiliations:** 1 Institute of Pathology, RWTH Aachen University, Aachen 52074, Germany; ekaemmerer@ukaachen.de; 2 Department of Pediatrics, RWTH Aachen University, Aachen 52074, Germany; 3 Institute of Pathology, Klinikum Braunschweig, Braunschweig 38114, Germany

**Keywords:** cancer, lipidation, modifier, Wnt

## Abstract

The wingless (Wnt) signaling is suggested as a fundamental hierarchical pathway in regulation of proliferation and differentiation of cells. The Wnt ligands are small proteins of about 40 kDa essentially for regulation and initiation of the Wnt activity. They are secreted proteins requiring acylation for activity in the Wnt signaling cascade and for functional interactivity with transmembrane proteins. Dual lipidation is important for posttranslational activation of the overwhelming number of Wnt proteins and is probably involved in their spatial distribution. The intestinal mucosa, where Wnt signaling is essential in configuration and maintenance, is an established model to study Wnt proteins and their role in carcinogenesis and cancer. The intestinal crypt-villus/crypt-plateau axis, a cellular system with self-renewal, proliferation, and differentiation, is tightly coordinated by a Wnt gradient. In the review, some attention is given to Wnt3, Wnt3A, and Wnt2B as important members of the Wnt family to address the role of lipidation and modifiers of Wnt proteins in intestinal carcinogenesis. Wnt3 is an important player in establishing the Wnt gradient in intestinal crypts and is mainly produced by Paneth cells. Wnt2B is characterized as a mitochondrial protein and shuttles between mitochondria and the nucleus. Porcupine and ACSL5, a long-chain fatty acid activating enzyme, are introduced as modifiers of Wnts and as interesting strategy to targeting Wnt-driven carcinogenesis.

## 1. Introduction

Wingless (Wnt) proteins are important mediators of cell proliferation and differentiation by activation of cell surface receptors. In Wnt responder cells, the Wnt signaling includes the canonical β-catenin pathway and several types of non-canonical pathway activities. This signaling is described as a non-canonical Ca^2+^ pathway and non-canonical planar cell polarity (PCP) pathway. Among the Wnt-related pathways, the canonical Wnt pathway is the best characterized signaling cascade and consists of hierarchical organized highly specialized proteins signaling extracellular events to the nucleus. By this mechanism, Wnt signaling is able to initiate transcriptional changes [1,2,3].

**Figure 1:**
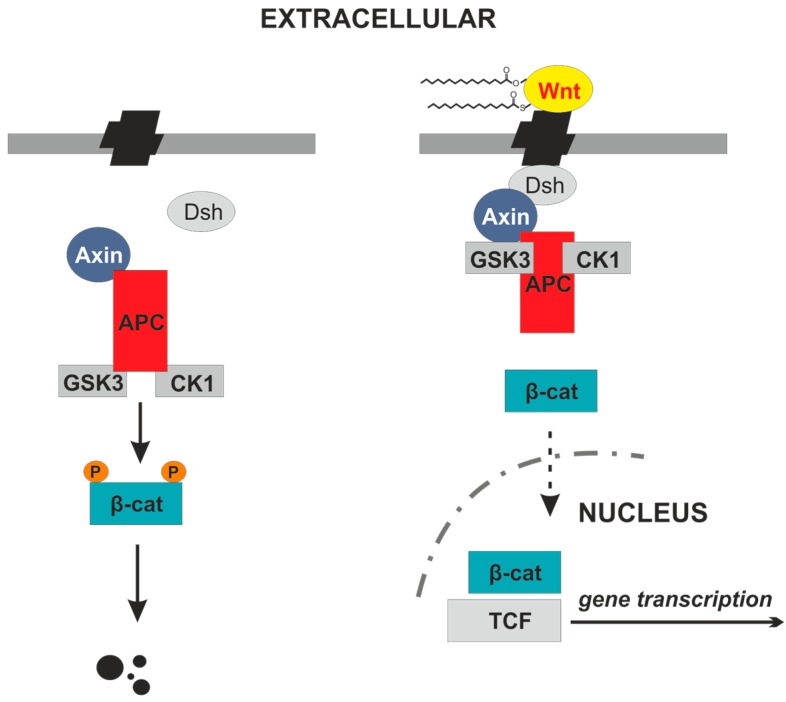
Scheme of canonical wingless (Wnt) protein signaling. (**Left**) In the absence of activated Wnt phosphorylation of β-catenin protein by a protein-kinase complex is found resulting in degradation of β-catenin; (**Right**) After coupling of activated Wnt to Fzd the protein-kinase complex is translocated and accumulation of β-catenin is found. The active β-catenin protein translocates into the nucleus and activates gene transcription via T cell factor (TCF) binding.

The central protein in canonical Wnt signaling is β-catenin which is able to translocate into the nucleus in its dephosphorylated state. Glycogen synthase kinase-3β (GSK-3β) acts in a complex with casein kinase 1 (CK1), axin, and adenomatous polyposis coli (APC) and is responsible for β-catenin phosphorylation and inactivation of the canonical Wnt-pathway [4]. In the presence of Wnt activity, β-catenin is dephosphorylated and has fundamental effects on gene transcription (Figure 1). Upregulation of the Wnt-cascade is found in stem cells and aberrant activation results in carcinogenesis in a variety of tissues and organs [5]. As mentioned above, additional pathways exist that bypass the Wnt-mediated nuclear translocation of β-catenin. For example, evidence is given for epidermal growth factor (EGF) and AKT-dependent phosphorylation of β-catenin promoting β-catenin transcription and nuclear accumulation that substitutes the canonical Wnt-pathway [6,7]. In addition, the calmodulin-dependent protein kinase II (CaMKII) is able to directly phosphorylate β-catenin at T332, T472, and S552 [8].

Secreted Wnt proteins, the most important inducers of Wnt signaling activities, control growth and patterning during development of multicellular structures, tissues, and organisms. It has been suggested that the important developmental function of Wnt proteins depends on the establishment of Wnt gradients, acting either as a short- or long-distance paracrine modifier of gene transcription [1]. The endogenous Wnt distribution in vertebrates is poorly understood and data concerning induction and maintenance of Wnt gradients are limited. Lipoprotein particles may partially counteract the palmitate-mediated affinity of Wnts for cell membranes, thereby allowing the spreading. However, Wnt-related transcriptional gene activity has been characterized in several tissue compartments including small intestinal mucosa crypts and several types of stem cells [2,9].

Since Wnt signaling is essential in several biological phenomena including proliferation and development of cells, organs as well as complex biological systems, increasing evidence is given that this important signaling pathway is involved in the pathogenesis of many types of diseases [10,11]. Intestinal and pulmonary carcinogenesis as well as different types of autoimmune diseases has been recognized as driven by Wnt signaling.

In this review, the current understanding of the emerging roles of Wnt proteins in intestinal carcinogenesis and cancer is given with a focus on Wnt lipidation. Wnt2B, Wnt3, and Wnt3A are discussed as models for Wnt family members to address lipidation and modifiers.

## 2. Wnt Proteins

In the evolutionary conserved Wnt pathway, induction of signaling is given by a small number of Wnt proteins. In humans, 19 Wnt genes that encode respective Wnt proteins have been identified so far [12,13]. Signaling induction is given by binding of the Wnt to the seven transmembrane spanning frizzled (Fzd) receptors. The Fzd receptors are also able to complex with distinct co-receptors such as the low-density lipoprotein-related receptor (LRP) 5 and 6. There is a diversity of pathway functions induced by the Wnt interaction with Fzd, because no special type of Wnt protein is restricted to a unique member of the ten Fzd family of proteins. The molecular diversity determines that the Wnt induced signaling activity is heterogeneous and includes activities in the canonical as well as non-canonical Wnt pathways such as the PCP and Ca^2+^ pathway. In addition, there are cross activities with other signaling cascades including JNK, RYK, CaMKII, and ROR [8,11,14].

The canonical Wnt-pathway is characterized by Wnt binding to its co-receptor complex that is substantiated with LRP-5 or -6. The signaling activity is further modified by intracellular proteins which influence extracellular antagonists such as secreted frizzled-related proteins (SFRP), the inhibitory factor WIF-1, and members of the dickkopf (DKK) family of secreted proteins. DKK1 and DKK3 are able to bind to LRP-5 and LRP-6 inhibiting the Wnt signaling by degrading the co-receptor [15]. In Figure 1, some key points of the Wnt-pathway including the β-catenin phosphorylation/dephosphorylation mechanism are shown.

As stated above the Wnt ligand type in addition to the expression domain and further determinants contributes essentially to the diversity and tissue specificity of Wnt functions. For example, Wnt16 has been found strongly associated with skeletal development and bone homeostasis [16], whereas Wnt3 is mainly synthesized by Paneth cells located at the bottom of small intestinal crypts [2]. The Wnts are about 350 amino acids long secreted cysteine-rich proteins that are modified by posttranslational mechanisms including lipidation and glycosylation. The Drosophila Wingless DWnt3/5 and DWnt4 are longer proteins with 1.004 and 539 amino acids respectively. The closest mammalian Wnt homologues are Wnt5 and Wnt9. However, the different Wnts generally have similar structural characteristics [17,18]. They have a molecular weight of about 40 kDa and contain several charged residues. Some of the cysteine residues are highly conserved between different species and involved in Wnt folding and multimerization [19]. Despite charged residues mammalian Wnt3A is a hydrophobic protein with high affinity to cell membranes. It has to be stressed that defined domains are not found in Wnts, but the sequence always includes a hydrophobic signal domain at the N-terminus that targets them to the endoplasmic reticulum (ER). In addition, a number of N-glycosylation sites exit in Wnt sequences.

## 3. Wnt Lipidation Is Essential for Secretion and Signaling Activity

Immature Wnts undergo strong posttranslational modifications in the ER including attachment of oligosaccharide chains in N-linked addition on the protein backbone. Some evidence is given that glycosylation makes protein secretion more efficiently and glycosylated Wnts are able to leave the ER. In the ER, the chaperone immunoglobulin heavy-chain-binding protein BiP, a member of the Hsp70 family, is able to associate with Wnt species and facilitate further sorting and final secretion of mature proteins, but can sequester defective Wnts in the ER [20]. The Wnt N-glycosylation is suggested as important signal for apical secretion and is not a modifier of Wnt folding and structure [18,21].

In addition to glycosylation, lipidation is a common feature of Wnt proteins and especially found in the N-terminal protein part. With the exception of WntD, Wnt proteins are posttranslationally modified by lipid adducts [11]. Specifically, the cysteine residue at position 77 (C77 in Wnt3A; C93 in Wingless, a Drosophila member of the Wnt family) is a highly conserved motive that is used as the structural basis for attachment of a palmitate group. The functional consequences of this lipidation are not fully elucidated as yet. It is speculated that the hydrophobic lipid group may promote Wnt trafficking through different cellular compartments and targeting to microsystems enriched in lipids. Specifically, Wnt lipidation is assumed as a targeting mechanism of Wnts to the ER and subsequent N-glycosylation. Other data argue for a structural modification preventing formation of aberrant disulfide bonds and protein retention in the ER and subsequent degradation [18]. Moreover, the palmitate moiety dependent hydrophobicity is in discussion to give the molecular basis for the spatial distribution of the Wnt morphogens with a local concentration gradient of a specific Wnt. The gradient distance includes up to 20 cell diameters around the source of Wnt production [22]. The model has been experimentally proofed for Wnt3A. Using a mutant variant of Wnt3A lacking the palmitoylated cysteine C77, overexpression was found as compensatory mechanism for diminishing local concentration and becomes more soluble in aqueous media [23]. It has been suggested that C77 palmitoylation might not be essential for direct canonical Wnt signaling activity but would rather be a prerequisite to concentrate the activated Wnt proteins at sensitive cell membranes. Furthermore, mutants of Wnt5a that have changed the cysteine C77 to alanine are secreted normally but are not able to induce a regular Wnt response [24]. In summary, the cysteine C77 could be characterized as the “lipidation for maturation/segregation”.

In addition to C77, which is conserved in most other Wnt proteins, another hotspot of lipidation, the serine residue at position 209 (S209), was revealed in Wnt3A. In contrast to C77, S209 is highly conserved among Wnts (Wnt8/WntD lacks the S209 equivalent) and modified with palmitoleic acid, a monounsaturated fatty acid [25]. The S209 modification is responsible for the strong hydrophobicity of Wnts, but the functional significance of this unusual fatty acid modification is not fully elucidated. Evidence is given for an anchoring function in cellular membranes by inserting into the lipid bilayer. Using a S209 mutant Wnt3A, the protein was retained in the ER and no longer secreted [26]. Consequently, the main function of the serine S209 could be addressed as “lipidation for secretion”.

As stated above hydrophobic activity of Wnts is due to their posttranslational addition of palmitate/palmitoleic acid to one or two residues. These lipid modifications of Wnt proteins are assumed as essential for the trafficking of secreted Wnts as well as Wnt recognition by Fzd receptors. It has been speculated that the Wnt acylation may directly engage the N-terminal Fz cysteine-rich domain and could also mediate binding to WIF-1 [26,27]. Regarding the lipid-related hydrophobic nature of secreted Wnt proteins special mechanisms are assumed for trafficking through the intracellular secretory pathway, release from cell membranes, and the extracellular transport assisted by lipoprotein particles, called argosomes [22,28,29]. Recently, the short-range Wnt gradient was visualized [30]. In the intestinal stem-cell niche, Wnt3 mainly travels away from its source in a cell-bound manner through cell division, and not through diffusion. In summary, the dual lipidation of Wnt proteins could be addressed as “lipidation for signaling activity”.

## 4. Porcupine and ACSL5 as Modifiers of Wnt Proteins

In the ER, the protein porcupine is found, which is a member of the membrane-bound O-acyltransferase family (MBOAT) and involved in N-glycosylation [19,31,32]. Porcupine is an eight transmembrane spanning protein ending within the ER membrane bilayer with the carboxy-terminal tail [33]. The molecular mechanism of Wnt glycosylation includes binding of the porcupine C-terminal region to an N-terminal domain with conserved cysteines. The binding to porcupine is probably essential in protection of the cysteine residues from forming disulfide bonds. Moreover, porcupine is assumed to target Wnt to the ER membranes and to give optimal access to the different glycosylation sites.

In addition to permit N-glycosylation, experimental evidence is given that porcupine catalyzes the lipidation of Wnts especially at S209 and equivalents resulting in hydrophobicity and membrane association [33,34]. The lipidated Wnts are transported from the ER to the transmembrane protein Wntless, which is associated with the Golgi apparatus. The Wnts are then shuttled by Wntless to the plasma membrane. It is suggested that the fatty acyl adduct of Wnts restricts the Wnt signaling to adjacent cells. The ER-resident stearoyl-CoA desaturases are involved in providing cellular palmitoleoyl-CoA by desaturation of palmitoyl-CoA. The functional association and participation of fatty acid metabolizing enzymes in the synthesis and modification of Wnt proteins is suggested as a molecular link coupling cell intrinsic fatty acid metabolism with the organization and coordination of cell and tissue homeostasis [35]. The chemical inhibition of desaturase enzymes is associated with a transcriptional up-regulation of many Wnt species. These experimental data demonstrating the important role of fatty acid metabolism to the Wnt activity and cell behavior [36].

Porcupine is essential in Wnt lipidation and no mechanism has so far been identified to fully compensate the enzymatic function [37]. Deletion of porcupine in mice results in an embryonic death due to defective gastrulation and disturbed secretion of Wnt proteins [38]. In primary human astrocytes and CD8^+^ T-cells, however, porcupine is not required for the release of Wnt proteins 1, 3, 5b, and other [39]. A special role of Wnt2B has been identified in these cells. While the inhibitor of porcupine, IWP-2, has no effect on the Wnt2B release, the siRNA-mediated porcupine knockdown reduces the Wnt2B secretion by 60 percent. In general, porcupine is able to recognize substrate and acyl donor specificity and exhibits some predilection for long chain fatty acids (about C10–C16 chain length).

Wnt proteins are mostly modified with palmitoate and plamitoleate. Thus, the relative cytoplasmic abundance of palmitoyl—and palmitoleoyl-CoA with other acyl-CoA species in addition to active site determinants and specialized domains of porcupine protein—Wnt protein interaction are assumed to translate and dictate enzyme selectivity [35]. This point of view is substantiated by the finding that acyl-CoA synthetase 5 (ACSL5), a long chain fatty acid activating enzyme, is able to promote Wnt palmitoylation [40].

ACSL5 is a nuclear-coded and mitochondrial expressed enzyme involved in the regulation of cellular apoptosis [41] and jejunal fatty acid activation [42]. In CaCo2 cells, ACSL5 dependent lipid profile changes are found with an increase of saturated acyl-CoA species after ACSL5 transfection, particularly C16:0, C18:0, C20:0, C22:0, C24:0, and C26:0, and hexosylceramides [43]. In ACSL5 KO mice the contrary is found. The animals display a decrease of 60 percent in jejunal palmitoyl-CoA synthesis rate [42].

**Figure 2:**
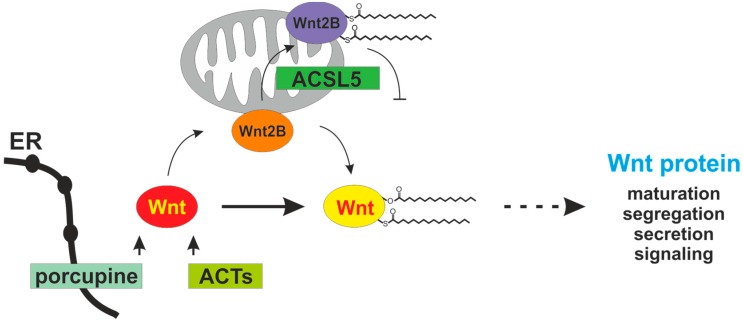
Scheme of Wnt lipidation and modifiers. The endoplasmic reticulum (ER)-associated acyltransferase porcupine is essential for Wnt lipidation with palmitoleic acid at S209 or equivalents and probably contributes to C77 palmitoylation. Porcupine is probably assisted by other acyl-CoA transferases (ACTs). Mitochondrial Wnt2B is arrested by ACSL5-dependent palmitoylation and does not further contribute to nuclear Wnt signaling.

The Wnt2B protein (also known as Wnt13) is a positive regulator of Wnt signaling [44]. The protein is synthesized by enterocytes and characterized by a shuttle mechanism between mitochondria and the nucleus [45]. The modifying capacity of ACSL5 on Wnt2B has been recently shown [40]. In mitochondria with ACSL5 overexpression (ACSL5^+^ mitochondria), the content of palmitoyl-CoA is significantly increased and strong plamitoylation of Wnt2B with intramitochondrial protein accumulation is found. The palmitoylated Wnt2B is not able to leave mitochondrial membranes and does not translocate into the nucleus for promoting the intranuclear Wnt signaling activities [40]. Porcupine and ACSL5 as Wnt modifiers are addressed in Figure 2.

## 5. Wnt Lipidation and Signaling in Intestinal Carcinogenesis and Cancer

The surface lining epithelial cells of the intestinal mucosa display a very high turnover rate with cellular replacement within a few days. Lineage-tracing studies and cell labeling experiments revealed that the intestinal crypt is the niche for proliferative, multipotent intestinal precursor cells. Interestingly, several key intestinal stem cell marker genes like Lgr5, Ascl2, and Sox9 are Wnt targets [46]. This is indicating for the requirement for strong Wnt signaling activity and an intact Wnt pathway in maintenance of the stem cell niche [2].

Similar to intestinal stem cells, cancer stem cells are suggested to give rise to progenitor cells populating the majority of an intestinal tumor. The Wnt is essentially involved in the establishment and proliferating activities of these stem cells. Two important concepts have been established describing intestinal carcinogenesis: the “top-down” and the “bottom-up” model. In both scenarios, Wnt signaling is considered as an important driver and regulator in the development of autonomy in tumor cell proliferation. In the “bottom-up” concept the stem cells at the crypt base acquire genetic alterations and migrate upwards constituting the tumor. The model has been substantiated by mice with conditional loss of APC in crypt base stem cells [47]. The loss of APC is associated with increased Wnt activity inducing transformation of epithelial cells located at the crypts. The contrary is suggested in the “top-down” model, where the differentiated surface lining epithelia re-acquire stem cell-like properties and expand into the crypts assisted by Wnt activities. In colorectal adenocarcinomas heterogeneity in Wnt signaling exists with aggressive Wnt^high^ cells and moderate Wnt^low^ cells [48,49]. Wnt lipidation could be an additional mechanism determining such differences in signaling activity. Signatures of Wnt protein lipidation characteristic either for the ‘top-down’ or the “bottom-up” carcinogenesis are not described so far.

In intestinal adenocarcinomas, heterogeneity of Wnt activity exists with increased proliferative activities in tumor cells with nuclear β-catenin expression. These cells are preferentially found at the invasive front of the tumor and apparently perform an epithelial- to -mesenchymal transition. The transition is suggested as important molecular and cellular mechanism to promote the malignant phenotype with establishment of tumor budding, drug resistance, and cancer stem cells [50]. Activation of Wnt signaling is associated with increased levels of Lgr5 in such adenocarcinomas. The Lgr5 expression correlates with the grade of malignity. Increased levels of Lgr5 were characteristic for higher stage tumors with strong local tissue infiltration and a high number of lymph node metastases [51]. Although Wnt signaling is able to strongly induce stem cell signature genes in tumor cells of colorectal adenocarcinomas, interactivity with other pathways is found to establish the stem cell behavior. One important co-signaling is given from NF-κB that regulates the pro-inflammatory cytokine response. Long-chain fatty acids, especially palmitate, are able to induce NF-κB activities and associated IL-6 expression [52]. The signaling cascade interacts with mitogen-activated protein kinase/Erk kinase (MEK), protein kinase C (PKC), and phosphoinositide-3 kinase (PI3K). Since triacsin C, an inhibitor of acyl-CoA activities including human ACSL5 [53], suppresses palmitate-induced NF-κB activation [52], long-chain fatty acids could be a molecular link by co-driving Wnt and NF-κB signaling. In addition, the inflammatory microenvironment displays a highly active NF-κB signaling promoting the acquisition of a stem cell-like behavior and neoplastic transformation [3,54,55]. In an APC deficient milieu, expansion of the Lgr5+ cancer stem cell compartment and initiation of carcinogenesis are found after the induction of NF-κB via Rac1 [56].

It is suggested that ACSL5 is involved in the early genesis of colorectal cancer, most likely by changing transport and pool formation of long-chain acyl-CoA thioesters [57]. In such adenocarcinomas frequent up-regulation of Wnt2B is found [58]. Up-regulation of both the putative modifier ACLS5 and the target Wnt2B points to a functional role in intestinal carcinogenesis. Using colon carcinoma cell lines of different malignity ACSL5-dependent plamitoylation of Wnt2B declines with an increase in malignity [40]. This finding suggests that the modifying role of ACSL5 to Wnt2B is more relevant in the early intestinal carcinogenesis than in manifest adenocarcinomas. The ACSL5-induced Wnt2B arrest in mitochondria is associated with a significant decrease in Wnt signaling activities. However, a systematic molecular characterization of Wnt lipidation in early intestinal carcinogenesis is not available. This is also true for other tumor entities like the NASH-associated hepatocellular carcinoma, where increased Wnt signaling is frequently found.

## 6. Therapeutic Modifiers of Wnt Lipidation

Modifiers of Wnt proteins and Wnt signaling are suggested as interesting molecular targets for anti-cancer strategies, because activation/dysregulation of Wnt signaling pathways are strongly involved in initiation and progression of neoplasias [48,59]. In particular, the modifiers of Wnt proteins coming more and more in the focus of distinct anti-tumor strategies. Much attention is given to targeting Wnt lipidation and the Wnt signaling at the “extracellular level” by the development of small molecules [60]. In carcinogenesis as well as stem cells, maintenance of homeostatic levels of desaturated phospholipids essentially for membrane fluidity is more reliable on de novo fatty acid desaturation than scavenging pathways [36,61].

For therapeutical intervention it is of importance that the modification of Wnt proteins at the two lipidation sites (C77 and S209 or equivalents) is apparently regulated differentially [33]. Whereas S209 lipidation occurs in a porcupine-dependent manner and regulates β-catenin mediated signaling, the C77 modification depends not exclusively on the porcupine activity and is involved in non-canonical Wnt signaling [33]. The acyltransferase responsible for C77 lipidation has not yet been fully characterized [59]. Despite different enzyme activities are apparently necessary to modify Wnt proteins with long-chain fatty acids, inhibition of the enzyme porcupine has been developed as a promising strategy to antagonize canonical Wnt signaling, because porcupine-mediated lipidation is essential for secretion and signaling activities of Wnt proteins [60,62,63]. Among the small molecules tested, LGK974 was very potent and specific to inhibit the enzyme and a clinical trial has been started [64].

Targeting Wnt protein lipidation is a promising strategy in the development of anti-cancer agents. Increasing evidence is given that lipidation is found in a variety of disease-related proteins which are not involved in carcinogenesis and cancer [59]. For example, myristoylation inhibitors are sufficiently in blocking the action of human pathogens like candida albicans, trypanosoma brucei, and leishmania major [65,66]. Farnesylation is required for activation of the tree major Ras isoforms H-Ras, N-Ras, and K-Ras4B [58]. In transgenic mice, the farnesyltransferase inhibitor lonafarnib is able to improve the progeria phenotype, a premature aging syndrome caused by a point mutation [67]. Finally, lipid-modified proteins are more and more established as sufficient biomarkers monitoring neoplastic and non-neoplastic diseases [68].

## 7. Conclusions

Among the different types of Wnt signaling, the canonical pathway is the best known and plays important roles in morphogenesis, cell development, and carcinogenesis. Wnt proteins, which are essential in initiating and promoting Wnt activities, are molecules with posttranslational modifications including intensive lipidation by modifiers like porcupine and ACSL5. Lipidation of Wnt proteins is essential for their maturation/segregation, secretion, and protein function. Within the canonical Wnt pathway, the activated Wnt proteins are essentially involved in carcinogenesis and cancer. Therefore, Wnt lipidation is suggested as an interesting strategy to target Wnt-driven carcinogenesis.
